# A combination of moderate and vigorous physical activities reduces the burden of multimorbidity: findings from Longitudinal Ageing Study in India

**DOI:** 10.1186/s41043-022-00323-9

**Published:** 2022-09-12

**Authors:** Sasmita Behera, Jalandhar Pradhan

**Affiliations:** Department of Humanities and Social Sciences, National Institute of Technology Rourkela, Rourkela, Odisha 769008 India

**Keywords:** Physical inactivity, Multimorbidity, Prevalence, Socioeconomic status, India

## Abstract

**Background:**

Physical inactivity is a significant factor contributing to the prevalence of non-communicable diseases (NCDs). The objective of this study is to examine the association between physical activity and multimorbidity among Indian adults aged 45 years and above by residence.

**Methods:**

Data from Longitudinal Ageing Study in India (LASI) 2017–2018, wave 1, a nationally representative study, are used to examine the above objective. A total of 59,073 adults aged 45 years and above are enlisted in the study. Physical activities and other demographic and socioeconomic variables have been used to describe the distribution of multimorbidity and investigate their relationship. Logistic regression is employed to examine the adjusted impact of physical activity on multimorbidity among Indian adults (45 + years) by residence.

**Results:**

The level of physical activity is inversely related to the prevalence of multimorbidity in India. The rate of multimorbidity ranges from 4 to 12% among moderately active individuals in rural areas, whereas it ranges from 9 to 34% in urban areas across the age groups of 45 to 75+ years. Notably, the individuals who engage in both moderate and vigorous activities have a lower prevalence of multimorbidity than those who engage in only moderate activities.

**Conclusion:**

Our study shows that physical inactivity has an association with the rise in multimorbidity in both rural and urban areas; however, the intensity of multimorbidity is higher in urban areas. The policymakers should consider the influence of moderate and vigorous physical activity as a key prevention measure of non-communicable disease and multimorbidity.

## Introduction

Non-communicable diseases (NCDs), such as cancer, diabetes, lung diseases, and cardiovascular diseases, are the leading cause of death in the world, with an estimated 41 million deaths annually, and 77% of all NCD deaths occur in the lower and middle-income country [1]. In India, for instance, 63% of total deaths were associated with NCDs in 2016 [2]. Most of these deaths might be averted by addressing the key risk factors of NCDs. In 2013, the World Health Organization (WHO) launched the global action plan for 2025, where it aims to reduce 25% of premature death from NCDs by working on the determinants of NCDs like tobacco use, alcohol consumption, physical activity, salt/sodium intake, blood pressure measurement, obesity, drug therapy, availability of the basic technology, and essential medicines [3].

Physical activity is considered as one of the major determinants of NCDs that needs to be focused on for non-communicable disease prevention and management [4–6]. It is estimated that in a year, four to five million deaths could be avoided if people become more active [7]. Physical activity prevents mortality and improves people’s strength, capacity to complete everyday tasks, participation in social interactions, mobility, cognitive performance, and life expectancy, all of which contribute to the overall quality of life [8]. Adherence to WHO recommendation on minimum physical activity level (i.e. 150 min/week) is associated with a 10% reduction in all-cause mortality [9, 10]. Despite the health-improving effect of physical activity, the global estimate shows that one in every four adults is not enough active physically [7] due to rapid industrialisation, technological advancement, and changing transport patterns [11].

Meeting the recommended physical activity is essential in preventing and managing specific NCDs like heart disease, diabetes, cancer, and hypertension [12, 13]. Concerning the effect of physical activity on multimorbidity, numerous studies are available from western countries [14–17]. However, the association between physical activity and multimorbidity remains inconclusive in the past studies, with some reporting statistically significant association [18, 19] while others reporting no significant association [14, 20–22].

Based on the above literature, the question arises how the frequency, intensity, and duration of physical activity needed for health benefits, particularly the effect of intensity (moderate vs. vigorous) on morbidity associated with NCDs in India. Moreover, the differences in the pattern of physical inactivity and multimorbidity that exists in both rural and urban areas have gone unexplored in the past literature. Growing urbanisation is accompanied by a sedentary lifestyle, obesity, and decreased physical activity, which are the key risk factors for increasing multimorbidity. Even in rural areas, there is a steady movement towards an urbanised lifestyle due to technology, the modern transport system, and the emergence of the mass media. So, it is crucial to comprehend the physical activity patterns in rural and urban India and their link with multimorbidity.

The present study attempts to fill the aforementioned gaps by examining the impact of physical activity, both moderate and vigorous intensity, on multimorbidity associated with major NCDs. This study included six chronic diseases because of their well-known connections to modifiable lifestyle risk factors, high prevalence, and their impact on health status and mortality. The hypothesis set for the test is whether being physically inactive is associated with an increased likelihood of multimorbidity. The objective is to study the pattern of the rural–urban difference in the prevalence of multimorbidity among Indian adults (> 45 years) and to examine the association between physical activity and multimorbidity among Indian adults aged 45 years and above by residence. Besides physical activity, the study also includes several socioeconomic and demographic covariates associated with the prevalence of multimorbidity.

## Methods

### Data source

Data used in the present study are derived from the Longitudinal Ageing Study in India (LASI), conducted by the International Institute for Population Science in collaboration with Harvard T. H. Chan School of Public Health (HSPH), University of Southern California (USC), and University of Washington, for 2017–2018. The objective of the study was to gather longitudinal data on the burden of disease, functional health, healthcare, and the social and economic well-being of older adults. The survey followed a multistage stratified cluster sampling design to collect the data in rural and urban areas. A rural area is the smallest habitation area in which the village normally follows the boundaries of a revenue village recognised by the district administration. Similarly, an urban area is defined as one with a population of at least 5000 people, and more than 75% of the male working population is non-agricultural, and a population density of at least 400 square kilometres [23]. Information was collected from 72,250 individuals aged 45 and above (irrespective of their spouses’ age) across all the states and union territories of India. However, the present study includes only 59,073 individuals (rural: 41,305; urban: 17,768) aged 45 and above for the analysis, for whom information was available on physical activity, demographic and socioeconomic characteristics, and the set of non-communicable diseases.

### Variables

#### Non-communicable disease multimorbidity

The dependent variable used in the model is the occurrence of multimorbidity. Multimorbidity is defined as the simultaneous occurrence of two or more diseases in the same individual [24–26]. In order to determine the presence of morbidity among the adults, a question was asked to the respondents. The question was: ‘Do you currently suffer from any of the listed diseases?’ Participants had given the option of a list of six chronic NCDs: diabetes, stroke, hypertension, lung disease, heart disease, and cancer, from which they selected the disease they suffered from. The self-reported prevalence of six diseases has been taken as the response variable, represented by a categorical variable with a value 1 if a person suffers from any of the above six NCDs, and 0, otherwise. The number of chronic diseases for each respondent was counted and classified as zero morbidity, one morbidity, or two or more morbidities.

#### Physical activity

The level of physical activity was assessed from the question, ‘How often do you engage in moderate or vigorous activities?’ The responses were measured on the Likert scale-like every day, more than once a week, once a week, one to three times a month, and never. In addition, the length of practicing these activities was also assessed from the question ‘How much time do you spend doing these physical activities’, and the responses are recorded in minutes. Based on the WHO physical activity recommendations, the participants are classified into different categories like moderate physical activity: those who are performing more than 150 min of moderate-intensity (cleaning the house, washing clothes, gardening, drawing water from a well) physical activity per week, vigorous physical activity: those who are performing more than 75 min of vigorous-intensity (jogging, exercise in the gym, swimming, heavy lifting, digging with a spade, fast bicycling) physical activity per week, and inactive: those who are doing neither moderate nor vigorous activity.

#### Socioeconomic and demographic covariates

Besides physical activity, other socioeconomic factors also affect multimorbidity. These are age (45–54, 55–64, 65–74, 75 above), residence (urban and rural), sex (male, female), marital status (currently married, divorced, others), and region (East, Central, Northeast, South, North, and West), level of education (no schooling, less than 5 years complete, 5–9 years complete, and more than 10 years complete), caste-like Scheduled Caste (SC), Scheduled Tribe (ST), Other Backward Caste (OBC), and others, living arrangement (living alone, living with a spouse, living with spouse and children, living with children and others, living with others only), religion (Hindu, Muslim, and others), wealth index (Poorer, Poorest, Middle, Richer, Richest), and obesity (yes/no).

### Statistical analysis

Descriptive statistics are used to show the prevalence of multimorbidity based on various socioeconomic and demographic factors. To measure the individual effect of each variable on multimorbidity, multivariable logistic regression is used after controlling for other potential confounders. All results were weighted. STATA version 16.0 software was used for the analysis.

## Results

Table 1 shows the socioeconomic and demographic characteristics of the study participants by residence. Out of 59,073 individuals, the majority of the population is from rural areas (70%), and only 30% are from urban areas. The percentage of older adults (> 65) are almost equally distributed in rural and urban strata. More than 50% of the study population are female, and people from the OBC category are more in rural and urban areas. Hindu religion constitutes more than 80% of the sample population. Around 74% of the participants are married, and only 19% have completed 5–9 years of education in the rural areas, while in the urban area, it is 72% and 25%, respectively. In both areas, around 36% of the people are not doing any physical activity, and 7% are doing only vigorous activity. Obesity is found to be more prevalent among urban (45%) samples than rural (19%). About 57% of individuals are living with their spouses and children.

**Table 1:** Characteristics of study participants by residence, LASI 2017–2018

Characteristics	Rural		Urban		All	
	*N*	%	*N*	%	*N*	%
*Age*	7133	18.44	4072	19.97	11,165	18.90
45–49	5997	15.51	3539	17.35	9488	16.06
50–54	5789	14.97	3143	15.41	8921	15.10
55–59	6206	16.05	2781	13.64	9051	15.32
60–64	5632	14.56	3057	14.99	8678	14.69
65–69	7921	20.48	3801	18.64	11,770	19.93
70+	7133	18.44	4072	19.97	11,165	18.90
*Gender*						
Male	18,051	46.67	8929	43.78	27,055	45.80
Female	20,629	53.33	11,464	56.22	32,018	54.20
*Wealth quintiles*						
Poorest	7959	20.58	4538	22.25	12,453	21.08
Poorer	8446	21.83	4070	19.96	12,565	21.27
Middle	8123	21.00	3866	18.96	12,043	20.39
Richer	7546	19.51	4079	20.00	11,611	19.66
Richest	6607	17.08	3840	18.83	10,401	17.61
*Caste*						
SC	8631	22.31	2592	12.71	15,802	26.75
ST	4196	10.85	644	3.16	11,475	19.42
OBC	17,008	43.97	9861	48.35	5042	8.54
Others	8844	22.87	7296	35.78	26,754	45.29
*Religion*						
Hindu	32,466	83.94	16,156	79.22	48,746	82.52
Muslim	3743	9.68	2894	14.19	6519	11.04
Others	2470	6.39	1343	6.59	3808	6.45
*Marital status*						
Currently married	28,891	74.69	14,656	71.87	43,621	73.84
Widowed	8865	22.92	4961	24.33	13,789	23.34
Divorced/separated/deserted/others	924	2.39	776	3.81	1663	2.81
*Education*						
No schooling	23,088	59.69	6070	29.76	29,943	50.69
< 5 years complete	4547	11.75	1991	9.76	6590	11.16
5–9 years	7230	18.69	5163	25.32	12,220	20.69
> 10 years	3815	9.86	7169	35.15	10,321	17.47
*Physical activity*						
Only vigorous	3061	7.92	1416	6.95	4503	7.63
Only moderate	11,780	30.49	8119	39.84	19,654	33.30
Both moderate and vigorous	10,188	26.37	3504	17.19	13,932	23.61
Inactive	13,610	35.22	7341	36.02	20,930	35.46
*Living arrangement*						
Living alone	1622	4.19	529	2.59	2193	3.71
Living with spouse	6724	17.38	2763	13.55	9588	16.23
Spouse and children	21,789	56.33	11,697	57.36	33,460	56.64
Children and others	7001	18.10	4465	21.89	11,367	19.24
Others only	1543	3.99	939	4.60	2465	4.17
*Overweight/obesity*						
No	31,244	80.78	11,141	54.63	43,072	72.91
Yes	7436	19.22	9252	45.37	16,001	27.09
*Region*						
North	4882	12.62	2602	12.76	7481	12.66
Central	9139	23.63	3017	14.79	12,388	20.97
East	10,754	27.80	2965	14.54	14,067	23.81
Northeast	1555	4.02	424	2.08	2030	3.44
West	5070	13.11	4527	22.20	9359	15.84
South	7279	18.82	6858	33.63	13,748	23.27
Total	41,305	100.00	17,768	100.00	59,073	100.00

Overall, 11.85% of adults aged > 45 are suffering from multimorbidity, with a higher prevalence in the urban population (19%) as compared to the rural population (8.77%) (Table 2). As expected, the prevalence of multimorbidity increases with age, and the highest prevalence is among the 65–74 years age group. Men (11.39%) and women (12.23%) are almost equally affected by multimorbidity. Individuals from higher wealth quintiles and other social classes are suffering more, 18.43 and 14.52%, respectively, from multimorbidity. The rate of multimorbidity is 21.91% among urban individuals who are not engaged in physical activity, compared to 11.45% among rural individuals. Similarly, the percentage of multimorbidity is higher in the case of obese individuals, with 16.25 and 24.28 in rural and urban areas, respectively. Divorced individuals are more suffering from multimorbidity (14.87%) than those who are currently married (11.03%). Individuals with more than 10 years of educational attainment have a higher percentage (17.42) of multimorbidity than those who have completed 5 years of education (13.03) or no formal education (8.77%). More individuals from the southern region are affected by multimorbidity, followed by the western part, both in the case of rural and urban areas.

**Table 2:** Prevalence of multimorbidity across socioeconomic characteristics by residence, LASI 2017–2018 (in %)

Characteristics	Rural	Urban	Total	Absolute difference (rural–urban)
*Age*				
45–49	3.38	8.13	4.89	− 4.75
50–54	7.11	10.09	8.07	− 2.98
55–59	7.88	18.97	11.28	− 11.09
60–64	10.62	22.46	13.79	− 11.84
65–69	10.86	28.73	16.34	− 17.87
70+	12.60	28.57	17.09	− 15.97
*Gender*				
Male	8.78	17.88	11.39	− 9.10
Female	8.77	19.87	12.23	− 11.10
*Wealth quintiles*				
Poorest	5.13	12.43	7.44	− 7.30
Poorer	6.99	14.98	9.25	− 7.99
Middle	8.15	17.62	10.80	− 9.47
Richer	10.82	23.12	14.58	− 12.30
Richest	13.87	28.03	18.43	− 14.16
*Caste*				
SC	7.38	15.95	9.07	− 8.57
ST	4.44	10.28	5.09	− 5.84
OBC	9.34	19.91	12.74	− 10.57
Others	11.09	19.62	14.52	− 8.53
*Religion*				
Hindu	8.31	18.95	11.38	− 10.64
Muslim	10.47	19.55	13.98	− 9.08
Others	12.27	18.36	14.14	− 6.09
*Marital status*				
Currently married	8.35	17.50	11.03	− 9.15
Widowed	10.39	24.67	14.87	− 14.28
Divorced/separated/deserted/others	6.43	11.02	8.3	− 4.59
*Education*				
No schooling	7.36	15.36	8.77	− 8.00
< 5 years complete	10.03	21.41	13.03	− 11.38
5–9 years	10.16	20.72	14.05	− 10.56
> 10 years	13.20	20.17	17.42	− 6.97
*Physical activity*				
Inactive	11.45	21.91	14.65	− 10.46
Only vigorous	7.16	16.50	9.72	− 9.34
Only moderate	9.19	19.06	12.74	− 9.87
Both moderate and vigorous	5.19	13.75	7.07	− 8.56
*Living arrangement*				
Living alone	10.58	17.68	12.07	− 7.10
Living with spouse	9.87	23.67	13.34	− 13.80
Spouse and children	7.89	16.15	10.40	− 8.26
Children and others	10.14	25.03	15.24	− 14.89
Others only	8.32	12.81	9.81	− 4.49
*Overweight/obesity*				
No	6.99	14.61	8.71	− 7.62
Yes	16.25	24.28	20.29	− 8.03
*Region*				
North	10.57	17.05	12.53	− 6.48
Central	5.38	12.23	6.84	− 6.85
East	8.02	18.74	9.99	− 10.72
Northeast	6.37	13.03	7.58	− 6.66
West	10.49	18.48	13.85	− 7.99
South	12.25	23.54	17.16	− 11.29
Total	8.77	19.00	11.85	− 10.23

**Fig. 1:**
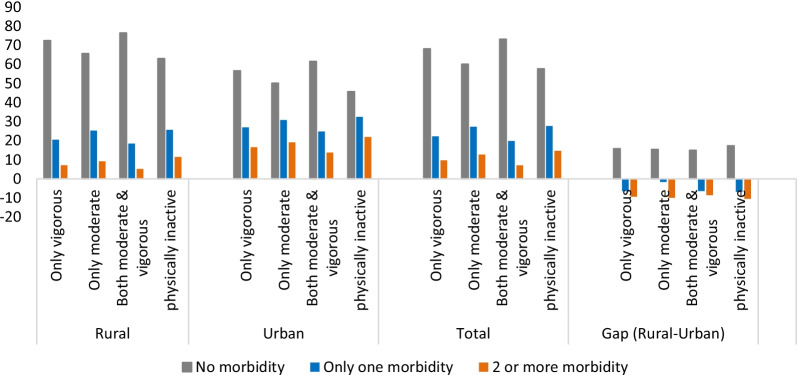
The status of physical activity and multimorbidity by residence

**Fig. 2:**
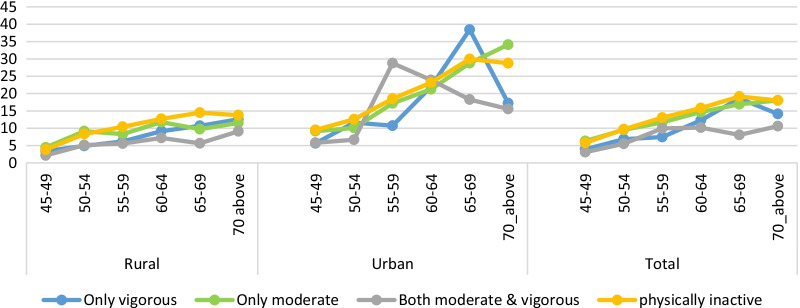
Age standardise prevalence of multimorbidity among physically active and inactive population by residence

Chronic NCDs are inversely related to physical activity in India. However, a large rural–urban difference is found in physical activity and multimorbidity. In urban areas, multimorbidity is higher than the rural area across all the categories of physical activities. On the other hand, the prevalence of multimorbidity is comparatively less among those individuals who participated in only vigorous activity or the combination of both moderate and vigorous activity as compared to those who are practising only moderate activity (Fig. 1). Likewise, age standardise prevalence of multimorbidity among physically active and inactive people shows that multimorbidity increases with age. Up to the age group of 65–69 years, the prevalence of multimorbidity shows an increasing trend, with the highest prevalence among physically inactive individuals. However, the multimorbidity rate decreases in the age group of people > 75 years compared to 65–69 years. The prevalence of multimorbidity ranges from 4 to 12% among individuals who are moderately active in rural areas. In contrast, it ranges from 9 to 34% in urban areas across the age group of 45 to 75+ years. Similarly, individuals doing both vigorous and moderate activity are less suffering from multimorbidity in both areas (Fig. 2).

The results from logistic regression analysis show that urban residents aged 65–74 years are more prone to have multimorbidity as compared to the 55–64 years age group, whereas in rural areas, the respondents aged 75 and above have the highest odds [3.16 (CI 2.46–4.05)] of multimorbidity as compared to all other age groups (Table 3). The prevalence of multimorbidity does not differ significantly in gender across rural–urban. As expected, multimorbidity increases with an increase in the income of the people. The likelihood of suffering from multiple chronic diseases is higher [OR 1.09 (CI 0.82–1.47)] among the Scheduled Caste population living in urban areas as compared to their counterparts. Widowed [OR in rural 1.05 (CI 0.65–1.67); OR in urban 1.54 (CI 0.77–3.06)] are more likely to suffer from multimorbidity compared to those currently married, divorced, or separated in both rural and urban population. Respondents with more than ten years of completed schooling suffer 1.90 times higher odds of morbidity than those without schooling. The probability of occurrence of multimorbidity is lesser among those who are physically active than in the physically inactive population in rural areas. In the urban area, the odds of multimorbidity are higher among those living with their spouse and children than those living alone. The obese respondents have a greater chance of suffering from multimorbidity as compared to the non-obese category. Similarly, the odds of multimorbidity are higher in the eastern, western, and southern regions than those of the northern, central, and northeast regions, both in the rural and urban areas.

**Table 3:** Odds ratio for multimorbidity by residence, LASI 2017–2018

Characteristics	Rural		Urban	
	OR	95% CI	OR	95% CI
*Age*				
45–49	1 [ref.]		1 [ref.]	
50–54	2.30*	[1.85–2.85]	1.28	[0.84–1.93]
55–59	2.71*	[2.21–3.32]	2.68*	[1.70–4.22]
60–64	3.94*	[3.22–4.82]	3.69*	[2.53–5.38]
65–69	3.96*	[3.22–4.87]	5.29*	[3.42–8.19]
70+	5.05*	[4.05–6.30]	5.43*	[3.45–8.53]
*Gender*				
Male	1 [ref.]		1 [ref.]	
Female	1.13***	[0.99–1.29]	1.08	[0.87–1.34]
*Wealth quintiles*				
Poorest	1 [ref.]		1 [ref.]	
Poorer	1.28**	[1.03–1.60]	1.17	[0.93–1.47]
Middle	1.41*	[1.15–1.73]	1.38**	[1.06–1.79]
Richer	1.80*	[1.48–2.19]	1.89*	[1.37–2.59]
Richest	2.13*	[1.75–2.59]	2.48*	[1.76–3.50]
*Caste*				
Others	1 [ref.]		1 [ref.]	
SC	0.92	[0.77–1.09]	1.13	[0.84–1.52]
ST	0.61*	[0.49–0.77]	0.73	[0.47–1.14]
OBC	0.97	[0.85–1.11]	0.98	[0.79–1.22]
*Religion*				
Hindu	1 [ref.]		1 [ref.]	
Muslim	1.32*	[1.12–1.55]	1.33**	[1.04–1.71]
Others	1.40*	[1.14–1.72]	1.03	[0.80–1.32]
*Marital status*				
Currently married	1 [ref.]		1 [ref.]	
Widowed	0.94	[0.59–1.51]	1.45	[0.73–2.88]
Divorced/separated/deserted/others	0.72	[0.39–1.30]	1.24	[0.59–2.62]
*Education*				
No schooling	1 [ref.]		1 [ref.]	
< 5 years complete	1.44*	[1.20–1.73]	1.45**	[1.07–1.96]
5–9 years	1.47*	[1.27–1.71]	1.49**	[1.08–2.05]
> 10 years	1.96*	[1.64–2.34]	1.31	[0.93–1.86]
*Physical activity*				
Inactive	1 [ref.]		1 [ref.]	
Only vigorous	0.65*	[0.52–0.81]	0.72	[0.46–1.13]
Only moderate	0.82*	[0.72–0.93]	0.87	[0.68–1.11]
Both moderate and vigorous	0.56*	[0.48–0.65]	0.73***	[0.51–1.05]
*Living arrangement*				
Living alone	1 [ref.]		1 [ref.]	
Living with spouse	0.91	[0.54–1.52]	2.68**	[1.20–5.99]
Spouse and children	1.03	[0.62–1.71]	2.66**	[1.18–6.03]
Children and others	1.09	[0.83–1.44]	1.97**	[1.12–3.49]
Others only	0.97	[0.69–1.38]	1.39	[0.78–2.46]
*Overweight/obesity*				
No	1 [ref.]		1 [ref.]	
Yes	2.25*	[1.97–2.56]	1.94*	[1.58–2.37]
*Region*				
North	1 [ref.]		1 [ref.]	
Central	0.66*	[0.54–0.81]	0.80***	[0.64–1.01]
East	1.04	[0.88–1.23]	1.34**	[1.06–1.68]
Northeast	0.82***	[0.66–1.01]	0.84	[0.60–1.18]
West	1.23**	[1.01–1.49]	1.11	[0.90–1.38]
South	1.41*	[1.20–1.66]	1.50*	[1.15–1.95]

*, **, *** indicates significant at 1%, 5%, and 10%, respectively

## Discussion

Based on nationally representative data, the present study reports the rural–urban difference in the prevalence of multiple chronic NCDs and how physical activity and the other socioeconomic covariates affect the prevalence of multimorbidity in India. As indicated by the present study, fewer people in urban areas are engaged in vigorous or a combination of both vigorous and moderate activity than rural residents, leading to a higher frequency of multimorbidity among urban adults. This finding concordance with a growing body of evidence showing that lack of physical activity is associated with multiple chronic conditions [15–17, 20, 27]. However, as suggested by many researchers, the rural–urban difference in physical activity is due to differences in occupational structure [28, 29]. Rural people are engaged in vigorous activities like farming, fishing, heavy lifting, and digging with a spade, but in urban areas, many work activities have been mechanised and automated, resulting in a more sedentary or inactive working environment. All of this, combined with higher pollution levels and westernised food habits, may be contributing to a higher occurrence of multimorbidity in urban areas [29, 30]. However, our result is consistent with previous investigations [28, 31] but in contrast to others that show that multimorbidity is higher among rural adults than in their urban counterparts [12, 30, 32].

Women, older adults, physically inactive individuals, and individuals with higher economic status have been identified as most vulnerable to multimorbidity irrespective of residence. It is found that the prevalence of multimorbidity is increasing up to the age group of 65–69 years, with physically inactive individuals having the highest prevalence. However, people over 75 years old have a lower multimorbidity rate than those aged 65–69. Here, the plausible reason could be that older people with several chronic diseases may have a low survival time; therefore, those who live for more than 75 years are likely to have fewer chronic diseases and lower multimorbidity. The age-related increase in multimorbidity is consistent with the findings from other studies [19, 28, 33, 34]. Rhodes et al. [35] found that older people exhibit poor self-efficacy in physical activity, thus perceiving fewer health benefits from doing so. Furthermore, poor health, such as heart, knee, or backache, arthritis, functional restrictions, loneliness, boredom, and fear of falling, also prevents older adults from engaging in physical activity, increasing the prevalence of multimorbidity [36]. A higher proportion of multimorbidity is reported by Muslims as compared to the Hindu religious group with no rural–urban differences. Similarly, individuals who belong to the ‘other’ social group are more likely to suffer from multimorbidity. However, after controlling for all other socioeconomic and demographic factors, no substantial caste difference in multimorbidity exists between rural and urban areas. Contrary to the other studies [29, 39–41], the present study shows that multimorbidity increases with an increase in education.

Aside from religion and social groups, significant socioeconomic and demographic differences are found in the prevalence of multimorbidity by residence. The current study directly links obesity and multimorbidity, as many prior studies have shown [16, 37, 38]. About 45% of the urban population having obesity in the present study is of considerable concern because of associated multimorbidity. The result of logistic regression shows that the people with obesity have a 1.90 times higher chance of developing multimorbidity than non-obese in urban areas. Although our findings reveal that rural areas have lower socioeconomic levels, such as lower levels of education and income, the impact of these socioeconomic variables on multimorbidity is greater in urban areas than in rural areas.

### Limitations

The present study is based on certain limitations. It is based on a cross-sectional design making it impossible to determine the observed directions of relationships among the variables. As both physical activity and prevalence of NCDs are measured by a questionnaire, self-reported errors might lead to an over-or underestimate of the outcome. The estimation of multimorbidity is based on a simplistic definition; a more comprehensive multimorbidity computation may yield different results. Despite the limitations, the findings of this study provide a detailed understanding of the level of physical activity on multiple chronic NCDs and other socioeconomic covariates among older adults in India. It could provide useful information for tracking the trends in physical activity among Indian adults and monitoring and evaluating efforts to stimulate physical activity and, consequently, reduce the burden of multimorbidity in India. Additionally, the study recommends improving the questionnaire by including a question on the reasons for not doing any physical activity, which could help better analyse the evolution of risk factors of multimorbidity.

## Conclusion

The findings of this study reveal that physical inactivity significantly contributes to several co-morbid conditions, which might have a negative impact on functional health and substantial life years lost. So, the detrimental effect of multimorbidity among older adults calls for a holistic approach to the management of these diseases, especially in urban areas. As urbanisation is unavoidable and is likely to accelerate in the future, appropriate lifestyle modifications must be pushed to mitigate its negative effects. According to the findings of the current study, those who engage in vigorous activity are less likely to develop NCDs multimorbidity than those who engage in moderate activity. So, as a policy recommendation, the government should emphasise encouraging active participation in vigorous activity. Recreational activities must be fostered in urban areas by providing facilities like gymnasiums, swimming pools, and playgrounds to promote vigorous physical activity. While establishing health initiatives for adults, vigorous physical activity or a combination of vigorous and moderate activities must be recognised as a strategy to reduce NCD-related comorbidities instead of only moderate activities. In addition, the most vulnerable groups (widowed, elderly aged 70+ years, and individuals of higher socioeconomic status) should receive more attention while formulating policies related to chronic multiple NCDs. Looking at the regional differences, as reported by our study, more priorities should be assigned to the eastern and southern Indian states to reduce the burden of multimorbidity.

## Data Availability

All the data are available on the website of International Institute of Population Sciences (https://www.iipsindia.ac.in/content/LASI-data).

## Abbreviations

NCDs: Non-communicable diseases
LASI: Longitudinal Ageing Study in India
WHO: World Health Organization
SC: Scheduled Caste
ST: Scheduled Tribe
OBC: Other Backward Caste
CI: Confidence interval
OR: Odds ratio
